# Biological constraints on GWAS SNPs at suggestive significance thresholds reveal additional BMI loci

**DOI:** 10.7554/eLife.62206

**Published:** 2021-01-18

**Authors:** Reza K Hammond, Matthew C Pahl, Chun Su, Diana L Cousminer, Michelle E Leonard, Sumei Lu, Claudia A Doege, Yadav Wagley, Kenyaita M Hodge, Chiara Lasconi, Matthew E Johnson, James A Pippin, Kurt D Hankenson, Rudolph L Leibel, Alessandra Chesi, Andrew D Wells, Struan FA Grant

**Affiliations:** 1 Center for Spatial and Functional Genomics, Children’s Hospital of PhiladelphiaPhiladelphiaUnited States; 2 Division of Human Genetics, The Children’s Hospital of PhiladelphiaPhiladelphiaUnited States; 3 Naomi Berrie Diabetes Center, Vagelos College of Physicians and Surgeons, Columbia UniversityNew YorkUnited States; 4 Department of Pathology and Cell Biology, Vagelos College of Physicians and Surgeons, Columbia UniversityNew YorkUnited States; 5 Columbia Stem Cell Initiative, Vagelos College of Physicians and Surgeons, Columbia UniversityNew YorkUnited States; 6 Department of Orthopaedic Surgery, University of Michigan Medical SchoolAnn ArborUnited States; 7 Division of Molecular Genetics (Pediatrics) and the Naomi Berrie Diabetes Center, Columbia University Vagelos College of Physicians and SurgeonsNew YorkUnited States; 8 Department of Pathology, The Children’s Hospital of PhiladelphiaPhiladelphiaUnited States; 9 Department of Pathology and Laboratory Medicine, Perelman School of Medicine, University of PennsylvaniaPhiladelphiaUnited States; 10 Department of Pediatrics, Perelman School of MedicineUniversity of PennsylvaniaPhiladelphiaUnited States; 11 Department of Genetics, Perelman School of MedicineUniversity of PennsylvaniaPhiladelphiaUnited States; 12 Division of Diabetes and EndocrinologyThe Children’s Hospital of PhiladelphiaPhiladelphiaUnited States; Icahn School of Medicine at Mount SinaiUnited States; Icahn School of Medicine at Mount SinaiUnited States

**Keywords:** bioinformatics, GWAS, functional genomics, Capture C, body mass index, waist-hip ratio, Human

## Abstract

To uncover novel significant association signals (p<5×10^−8^), genome-wide association studies (GWAS) requires increasingly larger sample sizes to overcome statistical correction for multiple testing. As an alternative, we aimed to identify associations among suggestive signals (5 × 10^−8^≤p<5×10^−4^) in increasingly powered GWAS efforts using chromatin accessibility and direct contact with gene promoters as biological constraints. We conducted retrospective analyses of three GIANT BMI GWAS efforts using ATAC-seq and promoter-focused Capture C data from human adipocytes and embryonic stem cell (ESC)-derived hypothalamic-like neurons. This approach, with its extremely low false-positive rate, identified 15 loci at p<5×10^−5^ in the 2010 GWAS, of which 13 achieved genome-wide significance by 2018, including at *NAV1*, *MTIF3*, and *ADCY3*. Eighty percent of constrained 2015 loci achieved genome-wide significance in 2018. We observed similar results in waist-to-hip ratio analyses. In conclusion, biological constraints on sub-significant GWAS signals can reveal potentially true-positive loci for further investigation in existing data sets without increasing sample size.

## Introduction

Genome-wide association studies (GWAS) have been widely employed to identify genetic variants that are associated with risk for disease and physiologically relevant traits (Tam et al., 2019; Visscher et al., 2017). GWAS was first described in 2005 (Klein et al., 2005) and has since been utilized to study a large range of common and complex traits in humans. As of July 2020, the GWAS Catalog consisted of 4628 publications reporting a total of 189,811 associations that achieved genome-wide significance (p<5×10^−8^) between genetic variants and human common complex traits. For instance, in 2009, 54 near-independent genome-wide significant (GWS) signals associated with variation in height had been identified in a population of tens of thousands of individuals (Visscher, 2008), while by 2014, that number had jumped to 697 and was estimated to explain more than 20% of the trait’s heritability (Wood et al., 2014). The number of significant GWS signals increased to 3,290 in 2018, accounting for nearly a quarter of the heritability (Yengo et al., 2018). And yet, despite these successes, GWAS has a clear shortcoming in that a large proportion of predicted heritability remains unexplained despite a constantly growing number of implicated loci (Tam et al., 2019). These novel loci are only identifiable using larger sample sizes, which come at a significant cost of both time and money, and each successive independent signal explains less and less of the overall estimated heritability (Zuk et al., 2012).

The additional signals achieving genome-wide significance in successive rounds of GWAS are often those that failed to achieve the commonly utilized p=5×10^−8^ threshold in initial rounds, a strict bar that can only be met when larger cohort sizes are recruited. Various approaches have been previously utilized to identify sub-threshold signals that go on to achieve genome-wide significance. Sub-threshold signals have been selected for replication in a stage 2 GWAS due to their proximity to putative candidate genes to identify novel loci, as with the identification of the obesity locus *NPC1* and replicated subsequently in a large meta-analysis (Meyre et al., 2009; Turcot et al., 2018). Epigenomic maps have also been utilized to implicate biologically relevant sub-threshold variants that were subsequently experimentally validated (Wang et al., 2016). We explored if it would be possible to apply a systematic genome-wide molecular-genetics-based approach to sub-threshold signals to predict single nucleotide polymorphisms (SNPs) that would go on to achieve genome-wide significance in a future round of GWAS. Over the past decade, sequencing technologies such aa RNA-seq (Wang et al., 2009), ATAC-seq (Buenrostro et al., 2013), and high-resolution promoter-focused Capture C Chesi et al., 2019; Hughes et al., 2014; Su et al., 2020 have been developed to facilitate the annotation of genes and their regulatory elements. Such data have been utilized to identify physical variant-to-gene interactions via three-dimensional genomics to implicate effector genes at GWAS loci where both sequence variant and gene reside within regions of open chromatin (Arnold et al., 2015; Çalışkan et al., 2019; Chesi et al., 2019; Cousminer et al., 2020; Javierre et al., 2016; Smemo et al., 2014; Su et al., 2019; Su et al., 2020). By leveraging high-resolution promoter-focused Capture C with ATAC-seq, it is possible to physically connect putatively functional non-coding elements, such as enhancers, harboring disease-relevant SNPs to promoters of specific genes thereby potentially mechanistically implicated in the SNP-associated phenotype. We hypothesized that by applying this variant-to-gene mapping method to sub-threshold SNP signals, the molecular constraints on these signals would result in a set of SNPs that are plausibly related to the biology of the trait in question, thereby bypassing the requirement of a larger GWAS to implicate these signals.

To test this hypothesis, we leveraged multiple GWAS data sets, increasing in sample size over time, to determine whether our sub-threshold implicated leads became genome-wide significant in a subsequent larger study. We chose to test this method utilizing GWAS data sets for body mass index (BMI) and waist-to-hip ratio adjusted for BMI (WHRadjBMI) as initial traits because they have both been the subject of particularly large GWAS efforts by the GIANT consortium. Additionally, both traits have implicated a large number of loci and therefore provide optimal statistical power for our purposes. With respect to variant-to-gene mapping, we utilized our existing 3D genomic data sets for adipocytes as well as hypothalamic neurons as previous studies have implicated these cell types in BMI-associated variants (Locke et al., 2015).

## Results

### Suggestive proxy SNP identification

We first applied our chromatin-based variant-to-gene mapping approach to predict BMI loci that would be subsequently reported as genome-wide significant in a 2015 GWAS (n = 339,224) but were only suggestive in 2010 (n = 249,796). Because the sentinel SNPs reported from a GWAS are not necessarily causal with regard to phenotype, but very likely in linkage disequilibrium (LD) with the responsible allele, we first identified proxy SNPs (r^2^ > 0.8) for each of the 2010 suggestive SNPs, 5×10^−8^≤p<5×10^−4^. Here, we detected 26,343 proxy SNPs; however, some of these proxy signals were in LD with an already established genome-wide significant signal. Given the purpose of this analysis was to identify new signals that were not yet genome-wide significant, we eliminated all proxies that were in even very modest LD, with an already genome-wide significant signal using an r^2^ > 0.1. This filtering left a residual of 23,197 signals.

### ATAC-seq and promoter-focused capture C

We generated ATAC-seq and promoter-focused Capture C libraries from mesenchymal stem cell (MSC)-derived adipocytes and leveraged our existing comparable data from human ESC-derived hypothalamic neurons to query open chromatin maps for open sub-threshold SNPs that contact open gene promoters (Pahl et al., 2020). The adipocytes were derived from mesenchymal stem cells. Three ATAC-seq libraries were sequenced and analyzed with the ENCODE pipeline (https://github.com/kundajelab/atac_dnase_pipelines). Peaks from all replicates were merged by bedtools (v2.25.0) provided peaks were present in at least two biological replicates. These resulted in 2,225,635 adipocyte peaks and 179,212 hypothalamic neuron peaks.

The adipose capture C libraries had an average of 1.4 billion reads per adipose library, with an average of 41% valid read pairs and 89% capture efficiency. We then called significant interactions using the CHiCAGO pipeline and performed analyses at a 1-fragment resolution to identify short-distance interactions (258,882 interactions) and at 4-fragment resolution to identify long-distance interactions (278,040) (Cairns et al., 2016).

### Variant-to-gene mapping reveals suggestive 2010 BMI loci that subsequently achieved genome-wide significance in 2015

We next identified physical contacts between any of the remaining proxy SNPs and gene promoters, with the additional constraint that both points of contact were within regions of open chromatin and that the SNP itself did not map to a gene promoter. This approach favors SNPs most likely to have a functional role in the regulation of genes relevant to a given trait and is therefore dependent on the cell type utilized to make such inferences. For BMI variant-to-gene mapping, we used data derived from MSC-derived adipocytes and human ESC-derived hypothalamic neurons because of the known roles of these cell types in BMI. To identify the point at which this approach is no longer viable due to noise, that is, at point of negligible return and excessive false positives, we stratified the suggestive regions into several bins: 5×10^−8^≤p<5×10^−7^, 5×10^−8^≤p<5×10^−6^, 5×10^−8^≤p<5×10^−5^, and 5×10^−8^≤p<5×10^−4^. Note that each successive bin is inclusive of the SNPs from the previous bin.

Upon identifying loci that passed these filters, we quantified those that had reached genome-wide significance by 2015. To avoid redundant inclusion of SNPs in LD with one another, we collapsed all biologically constrained SNPs into independent SNP clusters, designated here as separate independent ‘loci’. We defined such loci as the set of SNPs surviving the variant-to-gene mapping filter that were in LD with one another at a relatively relaxed r^2^ threshold of >0.4.

**Figure 1.**
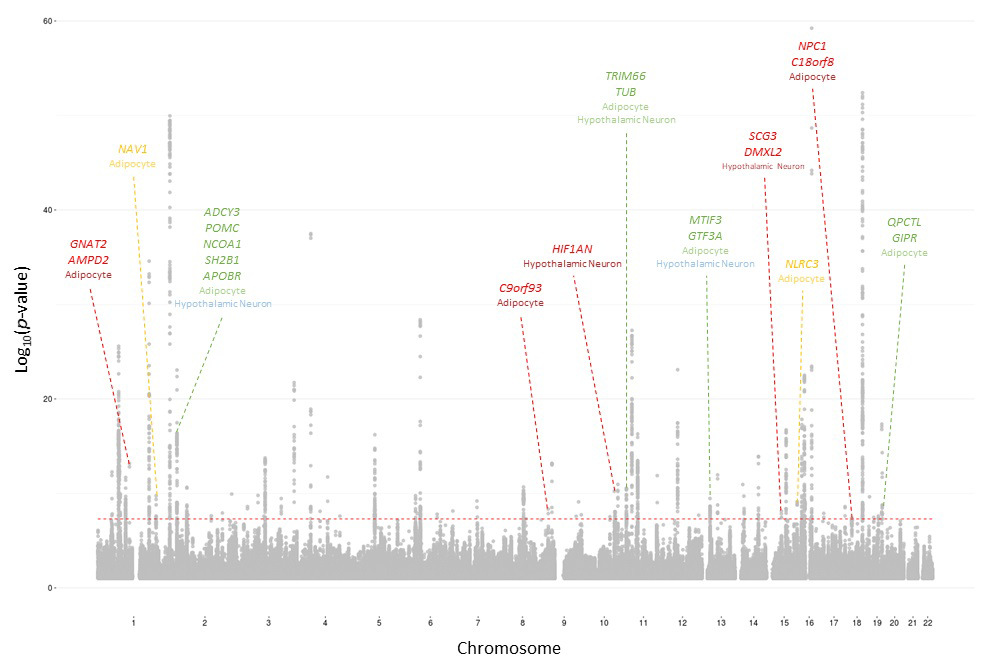
2015 BMI Manhattan plot depicting loci identified with 2010 salvaged SNPs 2015 BMI loci identifiable with 2010 salvaged SNPs. Cell type where locus was identified indicated below locus name. Color indicates the p-value threshold where the locus became implicated (Locke et al., 2015). Color key: Green – 5×10^−8^≤p<5×10^−7^, blue – 5×10^−7^≤p<5×10^−6^, orange – 5×10^−6^≤p<5×10^−5^, red – 5×10^−5^≤p<5×10^−4^.

**Figure 2.**
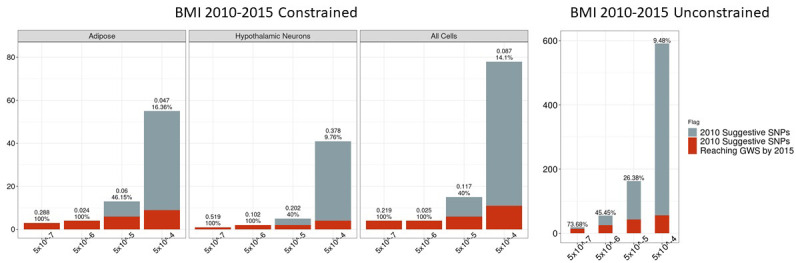
Independent 2010 BMI SNPs identified via variant-to-gene mapping that go on to reach genome-wide significance by 2015, as well as the set of unconstrained 2010 suggestive SNPs that achieve genome-wide significance by 2015. Positive predictive value is depicted as a percentage for each bar. Above these percentages, the p-value, as identified through Fisher’s exact test, is posted. These p-values depict the probability that the proportions of salvaged SNPs using variant-to-gene mapping differ from simply salvaging all suggestive SNPs within the same suggestive bin. Figure 2—source data 1.Number of 2010 loci identified by constrained method and the number that achieved GWS by 2015 in each cell type. Figure 2—source data 2.Number of 2010 loci identified with no constraint and the number that achieved GWS by 2015.

**Figure 3.**
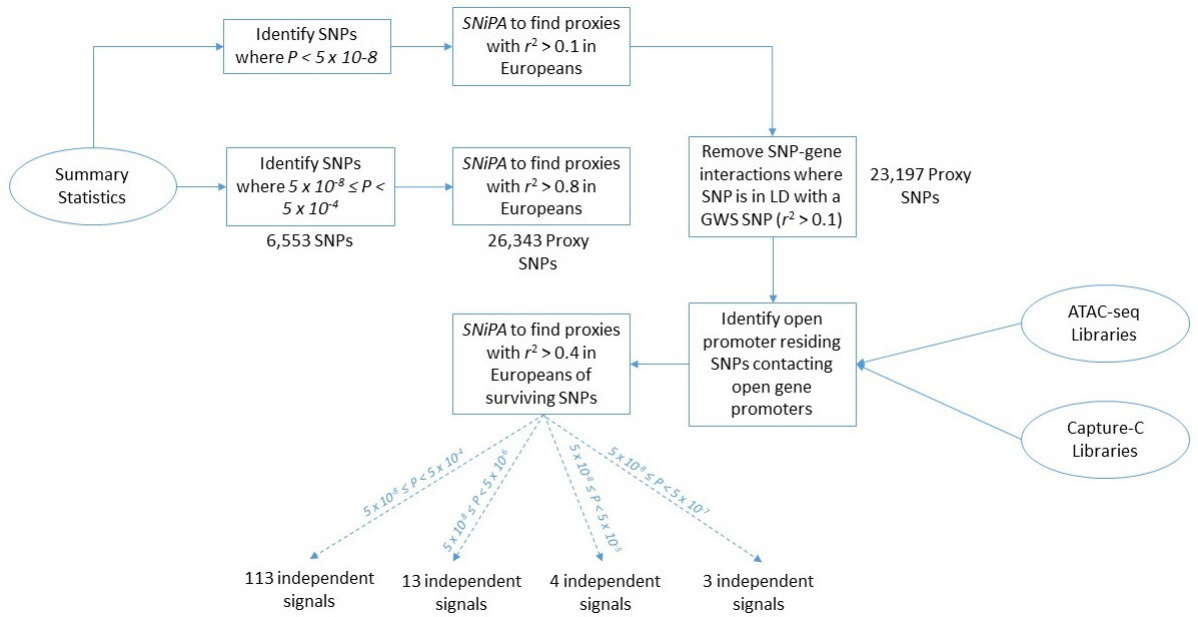
Flowchart of the pipeline describing each computational step. BMI 2010–2015 data is utilized here as an example to report the number of SNPs and loci that occur at each step of the analysis.

One hundred and sixty-one of the 23,197 suggestive proxy SNPs survived these biological filters at our most relaxed p-value threshold. These SNPs corresponded to 78 loci, of which 11 achieved genome-wide significance by 2015 (Table 1); these are annotated on the 2015 BMI Manhattan plot (Figure 1). Four of these loci were highlighted in the 2010 study, but the associations were only considered ‘suggestive’ within the stage one discovery set at that time, that is at p>5×10^−8^. Across all suggestive bins and cell types, the positive predictive value was low through 2015, and the proportion of constrained signals actually achieving genome-wide significance (GWS) did not differ significantly from the proportion of the unconstrained signals achieving GWS within the corresponding p-value bin (Figure 2). This is likely a function of the relatively modest increase in sample size (+89,428) between 2010 and 2015. At the relatively relaxed p-value threshold of 5×10^−8^≤p<5×10^−5^, we observed that 6/15 2010 biologically constrained loci were GWS in 2015, whereas 43/163 unconstrained loci were GWS by 2015. A flowchart describing this pipeline using the 2010–2015 BMI data is available in Figure 3.

**Table 1.** 2015 BMI loci that were implicated with our method in the 2010 data set. The 2015 genome-wide significant BMI loci whose sentinel SNP was in LD with SNPs implicated from suggestive association in the 2010 BMI GWAS. Key: Notable genes from biological relevance to obesity (B); copy number variation (C); DEPICT analyses (D); GRAIL results (G); BMI-associated variant is in strong LD (r^2^ ≥ 0.8) with a missense variant in the indicated gene (M); gene nearest to index SNP (N); association and eQTL data converge to affect gene expression (Q) (Locke et al., 2015).

Novel as of 2015 (Locke et al.)				
2015 sentinel SNP		2010 implicated SNPs	2015 assigned locus name	Interacting gene
rs4740619		rs10810462	*C9orf93*(C,M,N)	*TCONS_00015651*
rs17094222		rs117597828	*HIF1AN*(N)	*PAX2*
rs2820292		rs12086240, rs2820315	*NAV1*(N)	*TIMM17A*
rs758747		rs2238435	*NLRC3*(N)	*TCONS_00024950*, *TCONS_00024564*, *TCONS_00024949*, *TCONS_00024562*, *RP11-462G12.1*, *TCONS_00024568*, *TCONS_00024570*, *RP11-95P2.1*, *TCONS_00024567*, *TCONS_00024569*, *TCONS_00024320*, *TCONS_00024952*
rs3736485		rs7183479	*SCG3*(B,D); *DMXL2*(M,N)	*LYSMD2*, *SCG3*, *CTD-2308G16.1*, *TMOD2*
Identified between 2010 (Speliotes et al.) and 2015 (Locke et al.)				
2015 Sentinel SNP	2010 Implicated SNPs		2015 Assigned locus name	Interacting gene
rs17024393	rs72705210		*GNAT2*(N); *AMPD2*(D)	*GSTM3*, *AHCYL1*
rs4256980	rs10840079, rs10840087, rs11041999, rs11042023, rs12803166, rs4256980,		*TRIM66*(D,M,N); *TUB*(B)	*PBLD*, *RPL27A*, *TRIM66*
Identified in 2010 (Speliotes et al.), but was not genome-wide significant				
2015 Sentinel SNP	2010 Implicated SNPs		2015 Assigned Locus Name	Interacting gene
rs10182181	rs12713419, rs13012304, rs6718510, rs7597332, rs7608976		*ADCY3*(B,M,N,Q); *POMC*(B,G); *NCOA1*(B); *SH2B1*(B,M,Q); *APOBR*(M,Q);	*ADCY3*, *TCONS_00003602*
rs12016871	rs7988412,		*MTIF3*(N); *GTF3A*(Q) *~1 Mb from sentinel	*MTIF3*
rs1808579	rs1788783		*NPC1*(B,G,M,Q); *C18orf8*(N,Q)	*NPC1*
rs2287019	rs11672660, rs34783010		*QPCTL*(N); *GIPR*(B,M)	*GIPR*

### BMI GWAS variant-to-gene mapping constraints between 2010 and 2018

Using the same set of 2010 surviving SNPs from the previous section, we next identified how many of these SNPs reached GWS by the 2018 BMI GWAS (N = 681,275), representing a nearly tripling in cohort size compared to 2010. Here, we observed that 45 of the 78 2010 biologically constrained loci achieved genome-wide significance by 2018 at the most relaxed p-value threshold (Table 2).

**Table 2.** 2018 BMI loci that were identified using 2010 salvaged. SNPs 2018 BMI loci identified as genes nearest to genome-wide significant SNPs that could be identified using SNPs salvaged from suggestive regions of the 2010 BMI GWAS.

Nearest gene to sentinel	Surviving proxy SNPs	Lowest threshold found
*ABHD17A*	rs893543, rs893542, rs11671347	5 × 10^−4^
*AC007879.5*	rs11677847, rs72951700, rs11689163, rs72966483, rs11694560, rs11692026, rs964621, rs964622	5 × 10^−4^
*ADCY3*	rs6718510, rs7597332, rs7608976, rs13012304, rs12713419	5 × 10^−4^
*ADCY9*	rs710893, rs2531993, rs2238435	5 × 10^−5^
*AK5*	rs12729914	5 × 10^−5^
*AP000439.5*	rs11605729	5 × 10^−4^
*BCL7A*	rs7299842	5 × 10^−4^
*C10orf32*	rs7085104	5 × 10^−4^
*C18orf8*	rs1788826	5 × 10^−4^
*C1orf61*	rs11264483	5 × 10^−4^
*CCDC171*	rs10810462	5 × 10^−4^
*CNNM2*	rs1926032	5 × 10^−4^
*COQ4*	rs1468648	5 × 10^−4^
*CRTC1*	rs4808845, rs4808844	5 × 10^−4^
*DPYD*	rs12077442	5 × 10^−4^
*EIF2B5*	rs3914188, rs35637422	5 × 10^−4^
*EXOSC10*	rs1884429, rs12041740	5 × 10^−4^
*FAIM2*	rs422022	5 × 10^−4^
*GAB2*	rs869202	5 × 10^−4^
*GIPR*	rs34783010, rs11672660	5 × 10^−7^
*GPR61*	rs72705210	5 × 10^−4^
*HIF1AN*	rs117597828	5 × 10^−4^
*HOXB1*	rs2326013	5 × 10^−4^
*IFNGR1*	rs17258750	5 × 10^−4^
*IPO9*	rs2820315	5 × 10^−5^
*KCNJ12*	rs9906072	5 × 10^−4^
*LMOD1*	rs2047264	5 × 10^−4^
*MAP2K3*	rs2001651, rs3785542	5 × 10^−4^
*MAP3K7CL*	rs928277	5 × 10^−4^
*MEF2D*	rs2274319, rs1925950, rs12038396, rs3818463, rs2274320, rs2274317	5 × 10−^4^
*MLN*	rs11752353, rs6921487, rs72880511, rs1887340, rs73746509	5 × 10^−4^
*MLXIP*	rs28530689, rs10773037, rs28737311, rs36158849, rs2280573	5 × 10^−4^
*MST1R*	rs3774758, rs2252833, rs6446187	5 × 10^^−4^
*MTIF3*	rs7988412	5 × 10^−7^
*MTOR*	rs11581010, rs10864490	5 × 10^−4^
*NAV1*	rs12086240	5 × 10^−5^
*NPC1*	rs1788783	5 × 10^−4^
*RASA2*	rs2042864	5 × 10^−4^
*RCAN2*	rs3934393	5 × 10^−5^
*RNU6-543P*	rs10761689	5 × 10^−4^
*RP11-493K19.3*	rs13100903	5 × 10^−4^
*RP11-562L8.1*	rs12887636	5 × 10^−5^
*RP11-68I18.10*	rs10788800	5 × 10^−5^
*RP11-707P17.1*	rs7183479	5 × 10^−4^
*SAE1*	rs466477	5 × 10^−4^
*SKAP1*	rs16951519, rs2240121	5 × 10^−5^
*STK33*	rs10840087, rs11041999, rs34009921	5 × 10^−4^
*TNRC6B*	rs6001834, rs4820409	5 × 10^−4^
*TRIM66*	rs10840079, rs4256980, rs11042023, rs12803166	5 × 10^−6^
*TTC34*	rs6424062	5 × 10^−5^
*URM1*	rs7859557, rs2240948	5 × 10^−4^
*XXYLT1*	rs58434965	5 × 10^−4^

**Figure 4.**
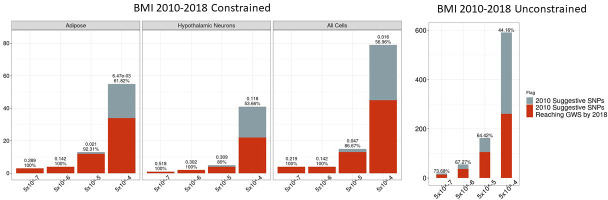
Independent 2010. BMI SNPs salvaged via variant-to-gene mapping that go on to reach genome-wide significance by 2018, as well as the set of unconstrained 2010 suggestive SNPs that achieve genome-wide significance by 2018. Positive predictive value is depicted for each bar. Above these percentages, the p-value, as identified through Fisher’s exact test, is posted. These p-values depict the probability that the proportions of salvaged SNPs using variant-to-gene mapping differ from simply salvaging all suggestive SNPs within the same suggestive bin. Figure 4—source data 1.Number of 2010 loci identified by constrained method and the number that achieved GWS by 2018 in each cell type. Figure 4—source data 2.Number of 2010 loci identified with no constraint and the number that achieved GWS by 2018.

The proportion of 2010 suggestive SNPs meeting these criteria that achieved GWS by 2018 within suggestive bin 5×10^−8^≤p<5×10^−5^ was particularly striking (Figure 4). At this threshold, 86.7% (13/15) of biologically constrained loci identified achieved genome-wide significance by 2018, whereas only 40% (6/15) had achieved genome-wide significance by 2015 (Figure 2); clearly, this improvement is a function of relative cohort size. While an increase was also observed with loci identified with no constraint (43/163 suggestive 2010 loci achieving GWS by 2015 and 105/163 achieving GWS by 2018), the precision was significantly higher with the biological constraint than without.

**Figure 4—figure supplement 1.**
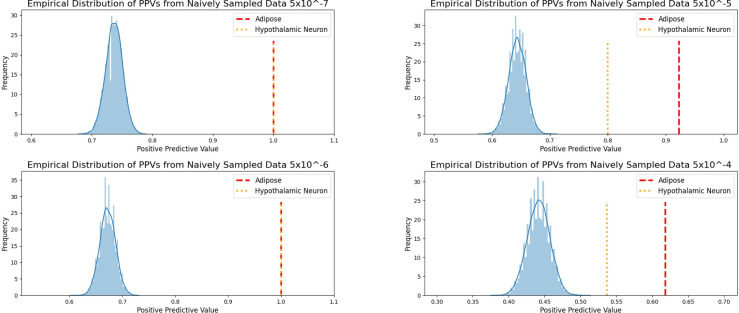
Empirical distribution of positive predictive values of suggestive 2010 BMI SNPs achieving GWS by 2018.

We assessed the positive predictive capability of this method across the stratified p-value bins to determine at which level of significance this biologically constrained analytic no longer outperformed randomly selected signals from the same bin. As shown in Figure 4—figure supplement 1, there is no point across either cell type or p-value bin where randomly sampled SNPs outperform the chromatin-constrained approach. Forty-four of 78 suggestive loci were identified in the 5×10^−8^≤p<5×10^−4^ bin, with a positive predictive value approximately 1.2-fold higher than random selection. As anticipated, fewer novel chromatin-implicated loci were detected in the 5×10^−8^≤p<5×10^−5^ bin, though the positive predictive value was approximately 1.5-fold higher than random. Taken together, these results suggest that this chromatin-constrained approach consistently performs better than random within these serial data sets, though there are diminishing returns as the p-value threshold is made increasingly less stringent.

### BMI GWAS variant-to-gene mapping constraints between 2015 and 2018

**Figure 5.**
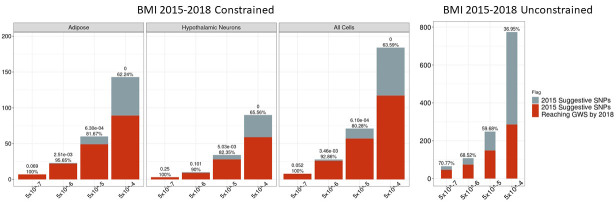
Independent 2015. BMI SNPs salvaged via variant-to-gene mapping that go on to reach genome-wide significance by 2018, as well as the set of unconstrained 2015 suggestive SNPs that achieve genome-wide significance by 2018. Positive predictive value is depicted for each bar. The posterior probability that loci identified by our chromatin-based constraint more often achieve GWS than loci with no constraint is posted above these percentages. Figure 5—source data 1.Number of 2015 loci identified by constrained method and the number that achieved GWS by 2018 in each cell type. Figure 5—source data 2.Number of 2015 loci identified with no constraint and the number that achieved GWS by 2018.

We also applied the chromatin-based variant-to-gene mapping approach to the 2015 BMI GWAS data to assess the efficiency of this analysis in identifying loci that would achieve genome-wide significance by 2018. The identified proxy SNPs, their nearest 2018 GWS proxy, and the nearest gene to those proxies are described in Supplementary file 1 (see Supplementary file 2 for the implicated genes). We observed that across all cell types with 5×10^−8^≤p<5×10^−4^, 117 of the implicated 184 loci reached GWS in 2018. At the 5×10^−8^≤p<5×10^−5^ threshold, 80% (57/71) of the constrained 2015 implicated loci reached GWS in 2018, while 59% (148/248) of the unconstrained 2015 loci reached GWS in 2018 (Figure 5).

The results from the analyses at these years present the greatest levels of significance observed thus far. Given the presence of many more loci in each bin, we observed significance at the 5×10^−8^≤p<5×10^−6^ threshold in both cell types, where 93% (26/28) of the surviving signals reached GWS by 2018 in both cell types. This appears to be principally due to the larger sample sizes at this bin size relative to 2010, which provided additional power to observe such differences. Considering the results of all BMI retrospective analyses through 2018, we found that this method positively identified sub-threshold BMI signals that went on to achieve GWS at a later date significantly more often than when we apply no biological constraint.

### Constraining WHRadjBMI GWAS reports: 2010–2018

**Figure 6.**
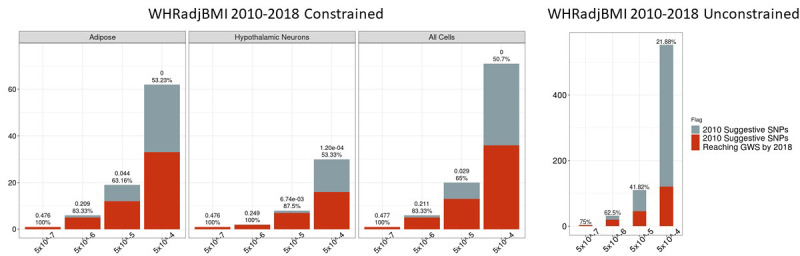
Independent 2010. WHRadjBMI SNPs salvaged via variant-to-gene mapping that go on to reach genome-wide significance by 2018, as well as the set of unconstrained 2010 suggestive SNPs that achieve genome-wide significance by 2018. Positive predictive value is depicted for each bar. Above these percentages, the p-value, as identified through Fisher’s exact test, is posted. These p-values depict the probability that the proportions of salvaged SNPs using variant-to-gene mapping differ from simply salvaging all suggestive SNPs within the same suggestive bin. Figure 6—source data 1.Number of 2010 loci identified by constrained method and the number that achieved GWS by 2018 in each cell type. Figure 6—source data 2.Number of 2010 loci identified with no constraint and the number that achieved GWS by 2018.

We also assessed the performance of this method in the 2010 WHRadjBMI GWAS (n = 77,167) relative to 2018 (n = 694,649) (The results of the WHRadjBMI 2010 WHRadjBMI relative to 2015 [n = 224,459,459], and 2015 WHRadjBMI relative to 2018, are available in our Figure 6—figure supplement 2, Figure 6—figure supplement 2, and Supplementary file 2). Here, we found 15,457 proxies within the most relaxed p-value threshold of 5×10^−8^≤p<5×10^−4^. One hundred and fifty-seven of these proxies survived our biological constraints, corresponding to 71 independent loci. Thirty-six of these ultimately achieved GWS by the 2018 GWAS (Figure 6).

**Figure 6—figure supplement 1.**
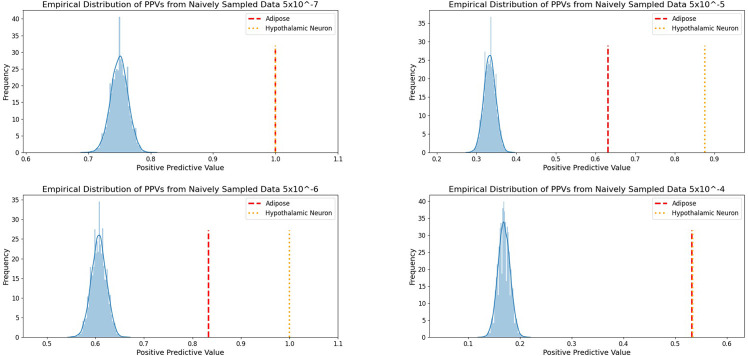
Empirical distribution of positive predictive values of suggestive 2010 WHRadjBMI SNPs achieving GWS by 2018.

**Figure 6—figure supplement 2.**
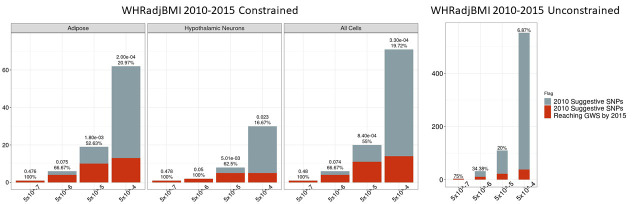
Independent 2010. WHRadjBMI SNPs salvaged via variant-to-gene mapping that go on to reach genome-wide significance by 2015, as well as the set of unconstrained 2010 suggestive SNPs that achieve genome-wide significance by 2015. Positive predictive value is depicted for each bar. The posterior probability that loci identified by our chromatin-based constraint more often achieve GWS than loci with no constraint is posted above these percentages. Figure 6—figure supplement 2—source data 1.Number of 2010 loci identified by constrained method and the number that achieved GWS by 2015 in each cell type. Figure 6—figure supplement 2—source data 2.Number of 2010 loci identified with no constraint and the number that achieved GWS by 2015.

**Figure 6—figure supplement 3.**
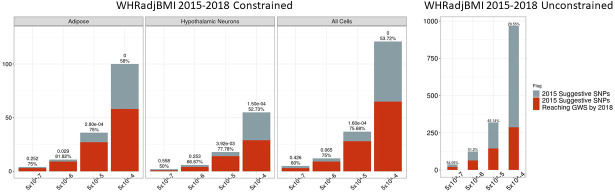
Independent 2015. WHRadjBMI SNPs salvaged via variant-to-gene mapping that go on to reach genome-wide significance by 2018, as well as the set of unconstrained 2015 suggestive SNPs that achieve genome-wide significance by 2018. Positive predictive value is depicted for each bar. The posterior probability that loci identified by our chromatin-based constraint more often achieve GWS than loci with no constraint is posted above these percentages. Figure 6—figure supplement 3—source data 1.Number of 2015 loci identified by constrained method and the number that achieved GWS by 2018 in each cell type. Figure 6—figure supplement 3—source data 2.Number of 2015 loci identified with no constraint and the number that achieved GWS by 2018.

Precision of the biologically constrained 2010 loci reaching genome-wide significance in 2018 for both traits through 5×10^−8^≤p<5×10^−6^ was 100% for each cell type, except for the 2010 WHRadjBMI SNPs constrained by adipose chromatin data which equals 83% (Figure 6—figure supplement 1). This represents a 1.3- to 1.6-fold increase over the mean PPV of a randomly sampled unconstrained SNP set at the same p-value threshold. The number of loci surviving this constraint present in this p*-*value threshold is quite modest (BMI: 4/4 vs WHRadjBMI: 5/6). The threshold may be further relaxed (5×10^−8^<p<5×10^−5^) to identify additional loci: BMI: 13/15 vs WHRadjBMI: 13/20. Relaxing even further to p<5×10^−4^ yielded far more loci, but brought in more potential false positives (BMI: 44/78 vs WHRadjBMI: 36/71), although they could still become GWS at a future time point. Despite the larger rate of false positives at a threshold of 5×10^−8^<p<5×10^−5^ in WHRadjBMI relative to BMI, we still observe that the chromatin-constraint surviving sub-threshold WHRadjBMI signals went on to achieve GWS at a later date significantly more often than when we apply no constraint.

### Predictive power of negative control does not differ from the unconstrained set

**Figure 7.**
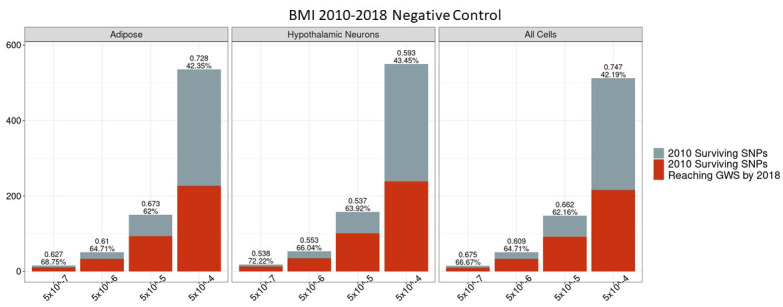
Independent 2010. BMI SNPs failing the variant-to-gene mapping filter that go on to reach genome-wide significance by 2018. Positive predictive value is depicted for each bar. The posterior probability that loci identified by our chromatin-based constraint more often achieve GWS than loci with no constraint is posted above these percentages. There is no threshold where this data differs significantly from the unconstrained set. Figure 7—source data 1.Number of 2010 loci identified by constrained method and the number that achieved GWS by 2018 in each cell type.

**Figure 8.**
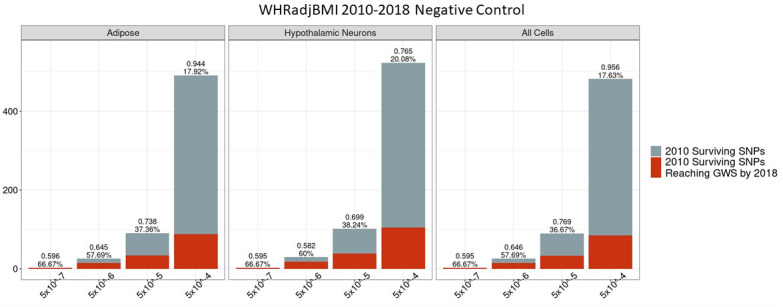
Independent 2010. WHRadjBMI SNPs failing the variant-to-gene mapping filter that go on to reach genome-wide significance by 2018. Positive predictive value is depicted for each bar. The posterior probability that loci identified by our chromatin-based constraint more often achieve GWS than loci with no constraint is posted above these percentages. There is no threshold where this data differs significantly from the unconstrained set. Figure 8—source data 1.Number of 2010 loci identified by constrained method and the number that achieved GWS by 2018 in each cell type.

We assessed whether there was any period (2010–2018) in which PPV for the set of SNPs that do not physically contact gene promoters or are not located within regions of open chromatin differed significantly from the unconstrained signals, that is, if there was any period in which such a negative control outperformed, the unconstrained signals. We found that there was no p-value bin, cell type or trait for which there was a difference between this negative set and the unconstrained set (Figure 7 and Figure 8). The absence of observable differences between the negative control and unconstrained sets supports the inference that the differences observed between our biological constrained data and the unconstrained data are in fact attributable to the chromatin-constrained analytic strategy.

Finally, we assessed the classification rates of this approach for both traits in all testable years. At each threshold for prior GWAS significance, we used our biological constraints to predict whether an implicated locus would achieve GWS in a subsequent GWAS. We found that, across all thresholds and chromatin-constrained loci in each cell type, sensitivity was generally less than 25%, indicating a high rate of false negatives. This is expected because the chromatin-constrained data represent effects that are only captured by the cell-types (and their respective developmental maturities) that were profiled. By combining the loci identified in either adipose or hypothalamic neurons, sensitivity was increased, though the increment was modest. Specificity, however, was consistently >80%, and often >90%, indicating few false positives. The precision was particularly high through 5×10^−8^<p<5×10^−5^; however, expanding through to 5×10^−8^<p<5×10^−4^ resulted in relatively high proportions of false positives in most analyses, although always at a lower rate than without a chromatin-constraint. As a control, we analyzed classifications of a negative control set in which loci failed to survive the variant-to-gene mapping filter for each cell type and suggestive threshold. There was no instance across trait, cell type, or p-value bin in which the specificity of the negative control outperformed the variant-to-gene mapping filter (Source data 1). All together, the consistently high specificity and precision observed conveys the capacity of this chromatin-constraint to preferentially retain signals that are likely to achieve GWS in larger data sets while minimizing the presence of false positives through 5×10^−8^<p<5×10^−5^.

## Discussion

In this study, we used our recently described variant-to-gene structural mapping approach (Chesi et al., 2019; Su et al., 2020) to conduct retrospective biologically constrained analyses of previous sequential GWAS reports to determine whether we could implicate statistically suggestive SNPs that would subsequently achieve GWS in incrementally larger data sets. Using a combination of high-resolution promoter-focused Capture C and ATAC-seq, this method enables the prioritization of statistically suggestive loci.

We assessed the extent to which this method can be applied to ‘salvage’ sub-threshold loci for possible consideration. We used BMI and WHR adjusted for BMI because of the relatively large number of loci identified in GWAS efforts conducted in large cohorts that continued to increase over time. The number of SNPs surviving these filters for each cell type and trait at each successively relaxed statistical cutoff are described in Source data 1.

Given the fairly modest increase in sample size between 2010 (N = 249,796) and 2015 (N = 339,224) for BMI, it was not particularly surprising that majority of the surviving 2010 BMI signals did not achieve GWS until 2018 (N = 681,275). Only six of the 15 surviving signals achieved GWS by 2015, but 13 eventually achieved GWS by 2018. Reaching deeper into the 2010 suggestive signals through p<5×10^−4^ showed a much clearer trend for both traits: roughly 20% of the surviving 2010 signals achieved GWS by 2015 and nearly 50% achieved GWS by 2018. Despite positive predictive values consistently greater than 80% for loci with 5×10^−8^≤p<5×10^−6^, it remains to be seen how many of the remaining surviving SNPs with less significant p-values will achieve GWS by the publication of the next GWAS in the future for each corresponding trait.

In this study, we never identified a particular sub-threshold bin where no constraint was as precise as this chromatin-based constraint. We did, however, find that the precision observed for loci in data sets between 2010 and 2018 varied by trait. While the positive predictive value was nearly 90% for 2010 BMI constrained loci reaching GWS by 2018 at 5×10^−8^≤p<5×10^−5^, only 65% of the surviving 2010 WHRadjBMI achieved GWS by the subsequent 2018 study. At the next, more restrictive, threshold, 5×10^−8^≤p<5×10^−6^, we found that nearly all of the surviving loci for both traits of the same years reached GWS by their respective 2018 study, although the number of loci within this threshold is small (BMI: 4/4, WHRadjBMI: 5/6). Thus, our findings suggest that this strategy is capable of identifying loci that will achieve GWS at 5×10^−8^≤p<5×10^−6^. Additional loci can be identified at 5×10^−8^≤p<5×10^−5^, at the expense of a degree of false positives. Although as noted above, such false-positive signals could possibly go on to be GWS in a future study.

We also observed that across all suggestive thresholds, specificity was consistently >80%, and often >90%. The consistently high specificity and precision, at least through 5×10^−8^≤p<5×10^−6^, suggest that true negatives are largely properly identified without the generation of large amounts of false positives. The low false-positive rate did come at a cost, however. Sensitivity was extremely low at all thresholds, often below 20% (Source data 1). The lack of sensitivity conveys high levels of false negatives, meaning many signals that would eventually achieve GWS would not be properly classified with this method.

The strength of this method is in its ability to attach biological relevance to sub-threshold SNPs in individual cell types, but this can also serve as its weakness. However, given the cost in time and resources to test candidate loci, we believe that high specificity and precision are more important characteristics for such a classifier. While we believe this trade-off is acceptable for the identification of novel biologically relevant loci prior to their confirmation via GWAS, we recognize that there are many loci that are falsely identified as not relevant. In utilizing the chromatin state of individual cell types in such a manner, we may reject loci that are biologically relevant in a different cell type or those that simply lack such an epigenetic mechanism. Provided a more comprehensive library of chromatin state in a much more diverse set of cell types, or the inclusion of additional biological filters that could identify loci that lack this epigenetic mechanism, we could potentially increase sensitivity in a substantial manner while retaining high rates of precision and specificity.

Despite any such limitations, we implicated loci most likely to become significant in the context of larger data sets with just our chromatin-based constraint approach. Using this variant-to-gene mapping approach, one can prioritize loci/genes of borderline statistical significance that may have important candidacy based upon functional considerations. Confirmation could come via larger data sets and/or by direct molecular physiological analyses of the candidates.

## Materials and methods

### ATAC-seq library generation and peak calls

Tn5 transposase transposition (Illumina Cat #FC-121–1030, Nextera) and purification of the Tn5 transposase derived DNA from 100,000 cells of each investigated cell type. The samples were then shipped to the Center of Spatial and Functional Genomics at CHOP where the ATAC-seq process was completed. Live cells were harvested via trypsinization, followed by a series of wash steps. One hundred thousand cells from each sample were pelleted at 550 × g for 5 min at 4°C. The cell pellet was then resuspended in 50 μl cold lysis buffer (10 mM Tris–HCl, pH 7.4, 10 mM NaCl, 3 mM MgCl_2_, 0.1% IGEPAL CA-630) and centrifuged immediately at 550 × g for 10 min at 4°C. The nuclei were resuspended in the transposition reaction mix (2× TD Buffer [Illumina Cat #FC-121–1030, Nextera], 2.5 µl Tn5 transposase [Illumina Cat #FC-121–1030, Nextera], and nuclease-free H_2_O) on ice and then incubated for 45 min at 37°C. The transposed DNA was then purified using the MinElute Kit (Qiagen), eluted with 10.5 μl elution buffer (EB), frozen, and sent to the Center for Spatial and Functional Genomics at CHOP. The transposed DNA was PCR amplified using Nextera primers for 12 cycles to generate each library. The PCR was subsequently cleaned up using AMPureXP beads (Agencourt), and libraries were paired-end sequenced on an Illumina HiSeq 4000 (100 bp read length) and the Illumina NovaSeq platform. Open chromatin regions were called using the ENCODE ATAC-seq pipeline (https://www.encodeproject.org/atac-seq/), selecting the resulting IDR conservative peaks (with all coordinates referring to hg19). We define a genomic region open if it has 1 bp overlap with an ATAC-seq peak.

### Cell fixation for chromatin capture

The protocol used for cell fixation was similar to previous methods (Hughes et al., 2014). Cells were collected and single-cell suspension were made with aliquots of 10^7^ cells in 10 ml media (e.g. RPMI + 10% FCS). Five hundred and forty microliters of 37% formaldehyde was added and incubated for 10 min at RT on a platform rocker. The reaction was quenched by adding 1.5 ml 1 M cold glycine (4°C) for a total volume of 12 ml. Fixed cells were centrifuged at 1000 rpm for 5 min at 4°C and supernatant removed. The cell pellets were washed in 10 ml cold PBS (4°C) followed by centrifugation as above. Supernatant was removed and cell pellets were resuspended in 5 ml of cold lysis buffer (10 mM Tris pH8, 10 mM NaCl, 0.2% NP-40 [Igepal] supplemented with protease inhibitor cocktails). Resuspended cells were incubated for 20 min on ice, centrifuged as above, and the lysis buffer removed. Finally, cell pellets were resuspended in 1 ml fresh lysis buffer, transferred to 1.5 ml Eppendorf tubes, and snap frozen (ethanol/dry ice or liquid nitrogen). Cells were stored at −80°C until they were thawed for 3C library generation.

### Capture C

Custom capture baits were designed using an Agilent SureSelect library design targeting both ends of DpnII restriction fragments encompassing promoters (including alternative promoters) of all human coding genes, noncoding RNA, antisense RNA, snRNA, miRNA, snoRNA, and lincRNA transcripts, totaling 36,691 RNA-baited fragments. The library was designed using scripts generously provided by Dr. Hughes (Oxford, UK), utilizing the RefSeq, lincRNA transcripts, and sno/miRNA tracks in the hg19 assembly downloaded from the UCSC Table Browser on 16 September 2015. The capture library design covered 95% of all coding RNA promoters and 88% of RNA types described above. The missing 5% of coding genes that could not be designed were either duplicated genes or contained highly repetitive DNA in their promoter regions.

The isolated DNA from the 3C libraries was quantified using a Qubit fluorometer (Life Technologies), and 10 μg of each library was sheared in dH_2_O using a QSonica Q800R to an average DNA fragment size of 350 bp. QSonica settings used were 60% amplitude, 30 s on, 30 s off, 2 min intervals, for a total of five intervals at 4°C. After shearing, DNA was purified using AMPureXP beads (Agencourt). Sample concentration was checked via Qubit fluorometer and DNA size was assessed on a Bioanalyzer 2100 using a 1000 DNA Chip. Agilent SureSelect XT Library Prep Kit (Agilent) was used to repair DNA ends and for adaptor ligation following the standard protocol. Excess adaptors were removed using AMPureXP beads. Size and concentration were checked again before hybridization. One microgram of adaptor ligated library was used as input for the SureSelect XT capture kit using their standard protocol and our custom-designed Capture C library. The quantity and quality of the captured library were assessed by Qubit fluorometer and Bioanalyser using a high-sensitivity DNA Chip. Each SureSelect XT library was initially sequenced on one lane HiSeq 4000 paired-end sequencing (100 bp read length) for QC. All six Capture C promoter interactome libraries were then sequenced three at a time on an S2 flow cells on an Illumina NovaSeq, generating ~1.6 billion paired-end reads per sample (50 bp read length).

### Analysis of promoter-focused capture C data

Quality control of the raw fastq files was performed with FastQC. Paired-end reads were pre-processed with the HiCUP pipeline (Wingett et al., 2015), with bowtie2 as aligner and hg19 as reference genome. Significant promoter interactions at 1-DpnII fragment resolution were called using CHiCAGO (Cairns et al., 2016), with default parameters except for binsize which was set to 2500. Significant interactions at 4-DpnII fragment resolution were also called with CHiCAGO using artificial .baitmap and .rmap files where DpnII fragments were grouped into four consecutively and using default parameters except for removeAdjacent that was set to false. We define PIR a promoter-interacting region, irrespective of whether it is a baited region or not. The CHiCAGO function peakEnrichment4Features() was used to assess enrichment of genomic features in promoter-interacting regions at both 1-fragment and 4-fragment resolution.

### Variant-to-gene mapping pipeline

BMI and WHRadjBMI GWAS summary statistics derived from European ancestry, each from 2010, 2015, and 2018, were downloaded from the Genetic Investigation of Anthropometric Traits (GIANT) consortium https://portals.broadinstitute.org/collaboration/giant/index.php/GIANT_consortium_data_files (Heid et al., 2010a; Locke et al., 2015; Pulit et al., 2019; Shungin et al., 2015; Speliotes et al., 2010b; Yengo et al., 2018). We identified all genome-wide significant variants from each data set as any SNP with p<5×10^−8^. We then identified the sets of variants in the varying suggestive bins of 5×10^−8^≤p<5×10^−7^, 5 × 10^−8^ ≤ p<5×10^−6^, 5 × 10^−8^ ≤ p<5×10^−5^, and 5 × 10^−8^ ≤ p<5×10^−4^. Upon the identification of these variants within each bin, we utilized *SNiPA* v3.3 (Arnold et al., 2015) to find their proxy SNPs, which we define as any SNP with r^2^ > 0.8 in Europeans for each region. Only signals with LD information within *SNiPA* were considered.

We next conducted a variant-to-gene mapping for each cell type within each of the suggestive bins to identify the gene promoters that are in contact with these SNPs. Promoter Capture C libraries are utilized to identify the genes with which these variants have interactions. Significant interactions were called using the CHiCAGO pipeline (Cairns et al., 2016) utilizing both 1-fragment and 4-fragment resolutions. We chose to focus on non-gene-to-gene interactions; thus, we ignored interactions that were identified as bait-to-bait as these represent the interactions between gene promoters. ATAC-seq libraries were also utilized to identify only those gene promoters that interact with SNPs in regions of open chromatin. Genes within the annotated MHC region were removed due to its highly polymorphic nature.

We next removed SNPs within the suggestive bins that were in linkage disequilibrium with any SNP that was significant genome wide. This was done to determine an entirely novel set of variant interacting genes that are entirely independent of those that are already known. To accomplish this, we removed all variants that were found to be in LD with a variant that is significant at a genome level. Thus, we identified all proxies of genome-wide significant variants at r^2^ > 0.1 and remove any variant within the suggestive region that is found to be a proxy of these genome-wide significant variants. This provides a filtered set of SNPs that are independent of any SNP that is significant at a genome-wide scale.

### Retrospective analysis

Interacting SNP-gene promoter pairs were identified by the variant-to-gene mapping pipeline described above for each GWAS across all suggestive zones for each cell type. Of these SNP-gene promoter pairs, we identify those that are in tight LD (*r*^2^ >0.8) with an SNP that is genome-wide significant in a future study. The implicated genes derived from our variant-to-gene mapping approach were frequently different from the locus ‘names’ traditionally used in publications reporting GWAS findings, typically based on the nearest gene. In contrast, our variant-to-gene mapping used the integration of ATAC-seq and promoter-focused Capture C data to identify the gene promotor(s) physically in contact with a genomic region (almost always non-coding) harboring an associated variant. However, in order to assess the 2015 loci that were identifiable in 2010 with this method, for consistency’s sake, we annotated the implicated 2010 loci with the published locus ‘name’ annotation from the respective 2015 GWAS report. However, unlike the 2015 efforts, the 2018 studies did not annotate the reported loci with an arbitrary gene notation; thus, we simply labeled the nearest gene to the 2018 sentinel SNP in which the surviving signals were in tight LD. The gene promoters in contact with each surviving signal are also available in Source data 1. The surviving SNPs that were in tight LD with an SNP that achieved GWS in 2015 were annotated with the published 2015 locus name of a sentinel SNP residing up to 1 MB away. We indicate the locus name that was provided in each 2015 study, as well as provide the set of gene promoters that were identified by the variant-to-gene mapping pipeline. Manhattan plots were generated in R (R Development Core Team, 2019), utilizing p-values of SNPs from the corresponding summary statistics. SNP positions were identified with dbSNP Build 151 to plot the relative location of the SNPs on the Manhattan plot. The highlighted loci point to the SNP with the lowest p-value identified at each locus.

In the case of all remaining comparisons, retrospective annotation was performed differently. Rather than trying to provide a locus name to the set of SNPs that were identified to be in tight LD with a genome-wide significant SNP in a later year, we elected to identify the surviving SNP, the gene promoters it physically contacts, and its best proxy that reached GWS (as defined by both r^2^ and p-value in the summary statistics).

To assess the predictive power of this method, we identified the positive predictive value of each set of salvaged SNPs across each retrospective analysis. To accurately quantify the number of loci that are identifiable using salvaged suggestive SNPs, we group the surviving variants based on their LD (r^2^ > 0.4) to one another as a locus. We extend this one additional time to include the proxies (r^2^ > 0.4) of each previously identified proxy SNP as a component of each locus. This allows SNPs that failed to be identified as a proxy of the lead SNP to be included within the locus via the transitive property, but only via one iteration of this process. The resulting loci identified are independent from one another, which prevents double counting of loci. We count these loci for each cell type across each suggestive bin, and we also identify the number of distinct loci regardless of cell type. We then identify the set of true positives as a locus that is in tight LD with a genome-wide significant SNP in a future GWAS. Those failing to meet these criteria are deemed false positives. We also identified loci of all genome-wide significant independent suggestive SNPs (r^2^ < 0.4) for each retrospective analysis and identified true-positive, false-positive, false-negative, and true-negative counts for this set using the same metrics as the constrained data sets. We used a beta-binomial model and used Monte Carlo approximation over 100,000 iterations to identify the posterior probability that loci identified by our chromatin-based constraint more often achieve GWS than loci with no constraint. We also assessed significance of the positive predictive values by calculating an empirical distribution from 10,000 iterations of a randomly sampled population of the unconstrained suggestive signals within each p-value threshold bin. At each iteration, we randomly selected N loci, where N is the corresponding number of loci surviving our chromatin-constraints from either cell type, and quantified the positive predictive value from this sampling. We then plotted the location where the positive predictive values fell for each cell type to determine whether these different significantly from the random population.

We calculated sensitivity, specificity, false-negative rate, and false positive rate of this approach across both traits for all testable years. To do this, we identified binary classifications of sub-threshold loci that were predicted to either reach or not reach GWS by the future year in each cell type separately. We then created confusion matrices for each cell type and p-value bin to calculate each binary classification performance metric. Additionally, we identified these values for the previously described negative control data (the effective inverse of the biological constrained data) as well as the randomly sampled data. For both of these data sets, we identified these metrics across 10,000 randomly sampled populations where we randomly selected N loci, where N is the corresponding number of loci surviving our chromatin-constraints from either cell type at the corresponding p-value threshold.

All source code available on Github: https://github.com/rkweku/SubThresholdProjectScripts.

## Data Availability

Adipose ATAC-seq and promoter-focused capture C data will be made available on GEO prior to publication. Hypothalamic Neuron ATAC-eq and promoter-focused capture C data is the subject of another atlas-based manuscript currently under peer review and through that process that dataset will be made available once the paper is published- the corresponding hypothalamus preprint can be found at: https://doi.org/10.1101/2020.07.06.146951v1.full. The following datasets were generated: HammondRKPahlMCSuCCousminerDLLeonardMELuSDoegeCAWagleyYHodgeKMLasconiCJohnsonMETerryNAGhanemLRBrownCSPippinJAHankensonKDLeibelRLChesiAWellsADGrantSFA2020MSC-Derived Adipose ATAC-seq and Promoter-Focused Capture-CNCBI Gene Expression OmnibusGSE164745 PahlMCDoegeCAHodgeKMLittletonSHLeonardMELuSRauschRPippinJABradfieldJPHammondRKBoehmKBerkowitzRILasconiCChesiAJohnsonMEWellsADVoightBFLeibelRLCousminerDLGrantSFA2020ESC-Derived Hypothalamic Neuron ATAC-seq and Promoter-Focused Capture-CNCBI Gene Expression OmnibusGSE164911 The following previously published datasets were used: SpeliotesEKWillerCJBerndtSIMondaKLThorleifssonGJacksonAUAllenHLLindgrenCMLuanJMagiR2010GWAS 2010 BMI Summary StatisticsBroad InstituteGIANT_BMI_Speliotes2010_publicrelease_HapMapCeuFreq HeidIMJacksonAURandallJCWinklerTWQiLSteinthorsdottirVThorleifssonGZillikensMCSpeliotesEKMagiR2010GWAS 2010 WHRadjBMI Summary StatisticsBroad InstituteGIANT_WHRadjBMI_Heid2010_publicrelease_HapMapCeuFreq LockeAEKahaliBBerndtSIJusticeAEPersTHDayFRPowellCVedantamSBuchkovichMLYangJCroteau-ChonkaDCEskoT2015GWAS 2015 BMI Summary StatisticsBroad InstituteSNP_gwas_mc_merge_nogc.tbl.uniq ShunginDWinklerTWCroteau-ChonkaDCFerreiraTLockeAEMagiRStrawbridgeRPersTHFischerKJusticeAEWorkalemahuTWuJM2015GWAS 2015 WHRadjBMI Summary StatisticsBroad InstituteGIANT_2015_WHRadjBMI_COMBINED_EUR PulitSLStonemanCMorrisAPWoodARGlastonburyCATyrrellJ2018GWAS 2018 BMI Summary StatisticsBroad InstituteMeta-analysis_Locke_et_al%2BUKBiobank_2018_UPDATED YengoLSidorenkoJKemperKEZhengZWoodARWeedonMNFraylingTMHirschhornJYangJVisscherPMGIANT Consortium2018GWAS 2018 WHRadjBMI Summary StatisticsZenodo10.5281/zenodo.1251813PMC648897330124842

## Additional files

Source data 1. Classification metrics for each condition.

Transparent reporting form
