# Supplementary material for: Biological constraints on GWAS SNPs at suggestive significance thresholds reveal additional BMI loci
Source: eLife. 2021 Jan 18;10:e62206. doi: 10.7554/eLife.62206 (PMC7815306; doi:10.7554/eLife.62206)
Supplement: Peer review history

# Decision letter

**DOI:** 10.7554/eLife.62206.sa1

**Affiliations:** Icahn School of Medicine at Mount SinaiUnited States; Canada

In the interests of transparency, eLife publishes the most substantive revision requests and the accompanying author responses.

**Acceptance summary:**

The study is well done and is likely to significantly impact our understanding gene associations in large GWAS datasets. Review critique was thoughtfully and thoroughly addressed. Notably, statistical correction for multiple testing of GWAS data requires increasingly large sample sizes to establish potential associations. This retrospective study used chromatin accessibility and direct contact with gene promoters as biological constraints. The application of such constraints on otherwise sub-significant GWAS signals was shown to reveal potentially true-positive loci without the requirement to increase sample size.

**Decision letter after peer review:**

Thank you for submitting your article "Biological constraints on GWAS SNPs at suggestive significance thresholds reveals true BMI loci" for consideration by *eLife*. Your article has been reviewed by two peer reviewers, one of whom is a member of our Board of Reviewing Editors, and the evaluation has been overseen by Clifford Rosen as the Senior Editor. The following individual involved in review of your submission has agreed to reveal their identity: David Meyre (Reviewer #2).

The reviewers have discussed the reviews with one another and the Reviewing Editor has drafted this decision to help you prepare a revised submission.

As the editors have judged that your manuscript is of interest, but as described below that substantial revisions are required before it is published, we would like to draw your attention to changes in our revision policy that we have made in response to COVID-19 (https://elifesciences.org/articles/57162). First, because many researchers have temporarily lost access to the labs, we will give authors as much time as they need to submit revised manuscripts. We are also offering, if you choose, to post the manuscript to bioRxiv (if it is not already there) along with this decision letter and a formal designation that the manuscript is "in revision at *eLife*". Please let us know if you would like to pursue this option. (If your work is more suitable for medRxiv, you will need to post the preprint yourself, as the mechanisms for us to do so are still in development.)

Summary:

The authors sought to determine whether SNP's located in chromatin that physically contacts GWAS hits provide evidence that boost test scores from suggestive to significant. If successful, this approach might obviate the need for larger, more expensive GWAS studies. The strategy is to first identify relevant open chromatin sites, focusing on genes that were suggestive in early studies. Then in parallel ask which suggestive loci in these early studies became significant in later and larger studies. They claim that positive results support the argument that epigenetic evidence would have boosted scores and precluded the need for larger and more expensive studies. The manuscript has potential, but work is needed. Critical evidence is not provided, or provided in a way that makes critical reading a challenge.

Essential revisions:

1) This is not the first study to demonstrate that biological annotations combined to more relax thresholds of statistical associations from GWAS “rescue” true associations in obesity field. As an illustration, Meyre et al., 2009, rescued a modest stage 1 GWAS association signal for extreme obesity in the NPC1 gene using a candidate gene strategy, and the association was replicated in stage 2. The GWAS obesity hit in NPC1 was recently confirmed as a genome-wide significant signal for BMI in a large meta-analysis (Turcot et al., 2018). Wang et al. (Diabetes 2019) demonstrated a strong enrichment in positive associations with BMI for SNPs located in/near syndromic obesity genes. The authors may like to discuss these and other reports in literature.

2) A strengths and limitation section would add a lot in the Discussion.

3) The biological criteria (chromatin accessibility / direct contact with gene promoters) used in this study are very original, but to be more exhaustive additional biological criteria may have been used to select more SNPs (listed in Li and Meyre, Int J Obes 2013 in the “hypothesis-driven GWAS analysis” section). This may be acknowledged as a limitation of the study.

4) Tissues targeted in the study (adipocyte and hypothalamus) are extremely relevant in the context of genetic susceptibility to obesity. However, genetic association studies have also highlighted the important role of other tissues in energy balance (e.g. beta-cells, liver, muscle, stomach… see Locke et al., 2015, and Pigeyre et al., Clin Sci 2016). Having not explored all tissues potentially relevant for obesity may be acknowledged as another limitation of the study.

5) While Speliotes et al., 2010 and Yengo et al., 2018 GWAS for BMI have been performed in populations of European ancestry, the Locke et al., 2015 study included a multi-ethnic population. Did the authors analyze the GWAS summary statistics in the European population in the Locke et al., 2015 study. If yes, they may provide more details on what they did in the Materials and methods section. If no, I think using the multi-ethnic GWAS summary statistics may add some heterogeneity, and I recommend to focus the analysis in the European population in the Locke et al., 2015 study.

6) Organization of data and analysis. Curiously, the epigenetic data, evidence and analysis of the Capture-C and ATAC-seq data is in the Materials and methods rather than the Results. The combination of methods and data are somewhat incomplete perhaps because of their unusual location in the manuscript. These are key to the overall study and should be in the Results section. As I read the manuscript, my curiosity grew about the nature of the evidence for connecting flanking and regulatory SNPs with the previously reported target SNPs – what is the source of the data, what is the nature of the evidence, – only to learn that they are in the Materials and methods, albeit incompletely. What is the evidence for open chromatin at their targets and in the sentinel genes that were suggestive in early GWAS studies?

7) Study design, analysis, and presentation. A flow chart would help readers understand the sequence of tasks and help the authors with the organization of the results.

8) Bottom-line. The key question is what is the benefit of the proposed method? What's missing – a simple statement about how many suggestive loci became significant later, and how many didn't, in the regular course of work, and in parallel how many epigenetic hits at suggestive loci become significant later, and how many didn't? That is, what's the quantitative benefit of the new assay. Remarkably they don't show the numbers for this seemingly simple and central question. Surprisingly, this point is not even in the Abstract; the 4th and 5th sentences, which address the key results, do not make these points clearly.

9) Chromatin evidence. The evidence is based on two cell lines – MSC-derived adipocytes and ESC-derived hypothalamic-like neurons. Both are relevant cell types in vivo. But beyond that functional connection, no other rationale is provided, and no discussion is provided to critically evaluate the reliability of the evidence. Chromatin states are dynamic properties of cells both in vivo and in vitro. Physiological conditions and disease state can impact these profiles. How stable are these profiles in vitro, and how consistent are these features with their in vivo counter-parts? How do these profiles vary among individuals in health or disease? What about single-cell heterogeneity? Presumably, these factors would contribute noise in the assays, creating false positives, false negatives. All of this is fine, in principle; every approach has limitations. But remarkably little consideration to these issues, either in study design or in discussion of the results and analysis. These issues need careful, thorough and critical consideration.

10) The authors should discuss how the model might work. The authors are correctly concerned about linkage disequilibrium, with an emphasis on independent evidence (subsection “Variant-to-gene mapping pipeline”). But no data are formally presented; if these SNPs are independent (no LD), what is the argument that these data can be combined rather than additive? Are positive results for this variant-to-gene mapping simply a reflection of additive effects? Or do they argue that SNP-interaction (epistasis) is involved? The text is unclear about these issues.

11) Approach. These are important questions for any proposed method:

A) The authors use a frequentist approach. Perhaps they justify that approach versus Bayes, which the logic of their approach nicely fits.

B) The authors should be able to give clear basic statistics – number/percentage of hits that validate in general, and then when applying their approach, essentially they need to give the false positive and false negative rates, i.e. how many does their approach put forward that end up being negative, and how many do they miss with their approach (presumably loci with non-epigenetic mechanisms would obviously be missed). Obviously, they need to be clear that a comparable threshold is being applied so that readers can assess the relative performance of the proposed and standard methods. Importantly, are they preserving the type I error rate with their new method, and what is their power? Unfortunately, the Abstract, Results and Discussion are not clear on these points – these are the essence of the paper.

# Reply: Author response

**DOI:** 10.7554/eLife.62206.sa2

Essential revisions:

1) This is not the first study to demonstrate that biological annotations combined to more relax thresholds of statistical associations from GWAS “rescue” true associations in obesity field. As an illustration, Meyre et al., 2009, rescued a modest stage 1 GWAS association signal for extreme obesity in the NPC1 gene using a candidate gene strategy, and the association was replicated in stage 2. The GWAS obesity hit in NPC1 was recently confirmed as a genome-wide significant signal for BMI in a large meta-analysis (Turcot et al., 2018). Wang et al. (Diabetes 2019) demonstrated a strong enrichment in positive associations with BMI for SNPs located in/near syndromic obesity genes. The authors may like to discuss these and other reports in literature.

The reviewer raises a good point and speaks to the feasibility of the uniform genome wide approach we have employed. In response to these points, we now refer to previous work in this context of using biological annotations to salvage sub-threshold signals. We’ve taken the suggestion of including the key example of the Meyre et al., 2009 study as an introduction to this idea of sub-threshold SNP mining, along with citing the Turcot et al. paper which replicated this association. We have also added an additional study by Wang et al., 2016, as it relates greatly to this paper in its use of epigenomic data to mine biologically relevant sub-threshold signals from GWAS.

2) A strengths and limitation section would add a lot in the Discussion.

We agree that a strengths and limitation section is an excellent addition to the manuscript. While we previously included a number of points regarding the limitations of this study, we recognize that a more complete breakdown is warranted. We have added a far more comprehensive discussion concerning the classification rates, noting the relatively poor sensitivity while also highlighting the high precision and specificity. We believe this trade-off is an acceptable outcome given the positive predictions went on to largely achieve genome-wide significance. As per comment #4, we have also noted in our limitations the lack of a more diverse set of cell types, although adipocytes and hypothalamic neurons are prime candidate, it is clear there are other relevant cell types that could be used in such a study; indeed they could be the subject of a future study in this regard.

3) The biological criteria (chromatin accessibility / direct contact with gene promoters) used in this study are very original, but to be more exhaustive additional biological criteria may have been used to select more SNPs (listed in Li and Meyre, Int J Obes 2013 in the “hypothesis-driven GWAS analysis” section). This may be acknowledged as a limitation of the study.

We agree that more criteria and constraints could always be employed. Our intention was to observe if this unique, and relatively straightforward, combination of chromatin state and physical contacts between implicated proxy SNPs and gene promoters alone would suffice to salvage sub-threshold SNPs. We do not doubt that additional filters could improve predictions to a degree, especially at more relaxed *p*-value thresholds, so we have highlighted this point in our Discussion while presenting the possibility that additional criteria could be used in a future study to improve the quality of the predictions.

4) Tissues targeted in the study (adipocyte and hypothalamus) are extremely relevant in the context of genetic susceptibility to obesity. However, genetic association studies have also highlighted the important role of other tissues in energy balance (e.g. beta-cells, liver, muscle, stomach… see Locke et al., 2015, and Pigeyre et al., Clin Sci 2016). Having not explored all tissues potentially relevant for obesity may be acknowledged as another limitation of the study.

We agree that more criteria and constraints could always be employed. Our intention was to observe if this unique, and relatively straightforward, combination of chromatin state and physical contacts between implicated proxy SNPs and gene promoters alone would suffice to salvage sub-threshold SNPs. We do not doubt that additional filters could improve predictions to a degree, especially at more relaxed *p*-value thresholds, so we have highlighted this point in our Discussion while presenting the possibility that additional criteria could be used in a future study to improve the quality of the predictions.

5) While Speliotes et al., 2010 and Yengo et al., 2018 GWAS for BMI have been performed in populations of European ancestry, the Locke et al., 2015 study included a multi-ethnic population. Did the authors analyze the GWAS summary statistics in the European population in the Locke et al., 2015 study. If yes, they may provide more details on what they did in the Materials and methods section. If no, I think using the multi-ethnic GWAS summary statistics may add some heterogeneity, and I recommend to focus the analysis in the European population in the Locke et al., 2015 study.

Thank you for catching this omission. We leveraged European ancestry results for all the studies analyzed. We have updated the Materials and methods accordingly to reflect this point.

6) Organization of data and analysis. Curiously, the epigenetic data, evidence and analysis of the Capture-C and ATAC-seq data is in the Materials and methods rather than the Results. The combination of methods and data are somewhat incomplete perhaps because of their unusual location in the manuscript. These are key to the overall study and should be in the Results section. As I read the manuscript, my curiosity grew about the nature of the evidence for connecting flanking and regulatory SNPs with the previously reported target SNPs – what is the source of the data, what is the nature of the evidence, – only to learn that they are in the Materials and methods, albeit incompletely. What is the evidence for open chromatin at their targets and in the sentinel genes that were suggestive in early GWAS studies?

Our intention with this form of presentation was to facilitate narrative flow and to dedicate largely the Results section to the process involving the retrospective analyses employed, while placing the processing of the epigenetic data in the Materials and methods section. However, given the reviewer’s comment, we agree that additional details should be provided in the Results section. We have therefore added additional information on these datasets in the Results section, while leaving the extensive details of how these data were generated and processed within the Materials and methods. The hypothalamic neuron related data were previously generated and analyzed in a separate study of ours – which we now cited. In addition, we include specific ATAC-seq peak numbers, as well as the interaction counts, for our three adipocyte libraries.

7) Study design, analysis, and presentation. A flow chart would help readers understand the sequence of tasks and help the authors with the organization of the results.

Given the complexity of these analyses, we strongly agree with this suggestion. To aid comprehension, we have now added a flowchart outlining the pipelines used (see Figure 3). The flowchart we include uses the BMI 2010-2015 data to exemplify our approach, and includes numbers to aid description of each computational step and how many SNPs and loci occur at each step of these analyses.

8) Bottom-line. The key question is what is the benefit of the proposed method? What's missing – a simple statement about how many suggestive loci became significant later, and how many didn't, in the regular course of work, and in parallel how many epigenetic hits at suggestive loci become significant later, and how many didn't? That is, what's the quantitative benefit of the new assay. Remarkably they don't show the numbers for this seemingly simple and central question. Surprisingly, this point is not even in the Abstract; the 4th and 5th sentences, which address the key results, do not make these points clearly.

We opted principally not to highlight these numbers outside of the various figures due to the many conditions that we were employed. This was to avoid the perception that we were somehow cherry-picking the best trait, year, and *P*-value bin given the variance in these numbers, potentially confusing or misleading the reader from the start. However, we do acknowledge that such numbers in text would be beneficial to keep the reader from consistently having to refer back to the figures and tables. Therefore, we have added such numbers at the end of each respective section using counts from within the consistent bin of 5 x 10^-8^ ≤ *P* < 5 x 10^-5^.

9) Chromatin evidence. The evidence is based on two cell lines – MSC-derived adipocytes and ESC-derived hypothalamic-like neurons. Both are relevant cell types in vivo. But beyond that functional connection, no other rationale is provided, and no discussion is provided to critically evaluate the reliability of the evidence. Chromatin states are dynamic properties of cells both in vivo and in vitro. Physiological conditions and disease state can impact these profiles. How stable are these profiles in vitro, and how consistent are these features with their in vivo counter-parts? How do these profiles vary among individuals in health or disease? What about single-cell heterogeneity? Presumably, these factors would contribute noise in the assays, creating false positives, false negatives. All of this is fine, in principle; every approach has limitations. But remarkably little consideration to these issues, either in study design or in discussion of the results and analysis. These issues need careful, thorough and critical consideration.

Our data are generated from three replicates and/or donors, and we focus on regions that are accessible across all replicates; indeed, we do note that there is high consistency between replicates. It should be noted that most chromatin differences across individuals are quantitative, not qualitative. A study by the Regev group (Gate et al., Nature Genetics 2018) showed that open chromatin regions in T cells from >100 individuals were highly consistent, but that 15% of OCR varied quantitatively in a manner associated with common genetic variation. Combined with the additional constraint of insisting on only SNPs coinciding with open chromatin via ATAC-seq, our Capture C based approach avoids a high degree of false positives; however, it is challenging to determine the false negative rate, given a lack of reference data. However, given the statistical significance of our observations, we strongly believe our data should be shared with the complex disease genetics field. Furthermore, we ensure we only work with cells that are derived from healthy individuals, given we are focused on characterizing the susceptibility conferred by these loci before onset of disease, i.e. we are investigating the genetic build up to disease pathogenesis, or in other words “before the car crash”. The data generated here are consistent with the datasets we describe in recent papers we published in Nature Communications for lupus and bone mineral density. Finally, we have not conducted chromatin conformation capture in a single cell setting, an approach which is still in its infancy and does represent some drawbacks, including important multiple amplification steps which can itself a high degree of false positives. We have highlighted these points in the Discussion.

10) The authors should discuss how the model might work. The authors are correctly concerned about linkage disequilibrium, with an emphasis on independent evidence (subsection “Variant-to-gene mapping pipeline”). But no data are formally presented; if these SNPs are independent (no LD), what is the argument that these data can be combined rather than additive? Are positive results for this variant-to-gene mapping simply a reflection of additive effects? Or do they argue that SNP-interaction (epistasis) is involved? The text is unclear about these issues.

We are less clear on the issues raised by the reviewer in this regard. We are in fact not concerned about LD, rather we are leveraging it to integrate with ATAC-seq data in order to shortlist putative causal variants, which are further shortlisted by the further integration of Capture C data. We are treating all the independent BMI and WHR loci as independent signals, and making inferences as such. Our study does not address or observe any form of epistasis.

11) Approach. These are important questions for any proposed method:

A) The authors use a frequentist approach. Perhaps they justify that approach versus Bayes, which the logic of their approach nicely fits.

We appreciate this suggestion. We have updated the statistical analysis with a Bayesian model to identify the probability that our constrained method outperforms no constraints. We note few changes to the results, though the situations where changes occurred resulted in >95% probability of our biological constraint outperforming no constraints. And overall, the changes do not affect our interpretation of these results.

B) The authors should be able to give clear basic statistics – number/percentage of hits that validate in general, and then when applying their approach, essentially they need to give the false positive and false negative rates, i.e. how many does their approach put forward that end up being negative, and how many do they miss with their approach (presumably loci with non-epigenetic mechanisms would obviously be missed). Obviously, they need to be clear that a comparable threshold is being applied so that readers can assess the relative performance of the proposed and standard methods. Importantly, are they preserving the type I error rate with their new method, and what is their power? Unfortunately, the Abstract, Results and Discussion are not clear on these points – these are the essence of the paper.

While we reported the sensitivity and specificity values in the previous version of the manuscript, we acknowledge that the false positive and false negative rates were not directly included. We did reference the general trend of these values, given their relationship to sensitivity and specificity, and the overall pattern of these rates across all analyses within the Results section entitled “Predictive power of negative control does not differ from the unconstrained set”, as well as in the Discussion; however, we acknowledge that such information relating to the preservation of the type I error rate was not included. To accommodate this comment, we have made our supplementary data file more comprehensive, which includes the classification metrics for each condition while also now including FPR and FNR. This file has been subsequently renamed Source data 1. Additionally, we now include a more direct discussion of these values and their trends within the Discussion section.
